# Gene Expression Profiling in Multiple Myeloma: Redefining the Paradigm of Risk-Adapted Treatment

**DOI:** 10.3389/fonc.2022.820768

**Published:** 2022-02-08

**Authors:** Claudio Cerchione, Saad Z. Usmani, A. Keith Stewart, Martin Kaiser, Leo Rasche, Martin Kortüm, María-Victoria Mateos, Andrew Spencer, Pieter Sonneveld, Kenneth C. Anderson

**Affiliations:** ^1^ Hematology Unit, IRCCS Istituto Scientifico Romagnolo per lo Studio dei Tumori (IRST) “Dino Amadori”, Meldola, Italy; ^2^ Division of Hematologic Malignancies, Memorial Sloan Kettering Cancer Center, New York, NY, United States; ^3^ Princess Margaret Cancer Centre, University Health Network, Toronto, ON, Canada; ^4^ Division of Molecular Pathology, The Institute of Cancer Research, London, United Kingdom; ^5^ Department of Haematology, The Royal Marsden Hospital, London, United Kingdom; ^6^ Department of Internal Medicine II, University Hospital of Würzburg, Würzburg, Germany; ^7^ Department of Hematology, University Hospital of Salamanca, Salamanca, Spain; ^8^ Malignant Haematology and Stem Cell Transplantation Service, Alfred Hospital-Monash University, Melbourne, Australia; ^9^ Department of Hematology, Erasmus MC Cancer Institute Rotterdam, Rotterdam, Netherlands; ^10^ Jerome Lipper Multiple Myeloma Center, Department of Medical Oncology, Dana-Farber Cancer Institute, Boston, MA, United States

**Keywords:** multiple myeloma, gene expression profiling (GEP), risk-adapted treatment, novel agents, SKY92, risk stratification

## Abstract

Multiple myeloma is a blood cancer characterized by clonal proliferation of plasma cells in the bone marrow. In recent years, several new drugs have been added to the therapeutic landscape of multiple myeloma, which have contributed to increased survival rates. However, while the use of therapeutics has evolved, there is still a group of high-risk patients who do not benefit from current treatment strategies. Risk stratification and risk-adapted treatment are crucial to identify the group of patients with urgent need for novel therapies. Gene expression profiling has been introduced as a tool for risk stratification in multiple myeloma based on the genetic make-up of myeloma cells. In this review we discuss the challenge of defining the high-risk multiple myeloma patient. We focus on the standardized analysis of myeloma cancer cells by gene expression profiling and describe how gene expression profiling provides additional insights for optimal risk-adapted treatment of patients suffering from multiple myeloma.

## Introduction: Risk Stratification in Multiple Myeloma

Multiple myeloma (MM) is a blood cancer characterized by clonal proliferation of plasma cells in the bone marrow (1). MM accounts for 1.2% of all cancers and 17.1% of blood cancers in Europe, North America, Australia and New Zealand. In these countries, 80,498 patients were newly diagnosed in 2018. In addition, the global prevalence of MM in 2018 was estimated at 159,985.^1^ MM is more common in the elderly, with a median age at diagnosis of 70 years. As a consequence, the number of myeloma patients is expected to rise as it follows the ageing population worldwide (2).

In the last 10 years, new therapies and novel mechanisms of action have been introduced in the clinical landscape of MM. The inclusion of immunomodulatory drugs (e.g. lenalidomide, thalidomide and pomalidomide), proteasome inhibitors, (e.g. bortezomib, carfilzomib) and therapeutic monoclonal antibodies (e.g. daratumumab, isatuximab) led to a significant improvement in survival (3). The median overall survival for newly diagnosed patients treated with high-dose therapy is between 4 and 10 years (4). Although some patients may have long term remission or “functional cure”, MM is a chronic relapsing disease for the majority of patients. By combining up to 4 drugs (*quadruplet regimens*) with different mechanisms of action, the list of possible treatment regimens is endless, creating a complex decision tree in the clinical path for a newly diagnosed patient (5).

Despite the enormous progress that has been made in prolonging survival in MM, there is a fraction of patients who do not respond to any of the available treatments or relapse rapidly after an initial response and have reduced survival. In literature, these patients are referred to as *high-risk* patients. The definition of high risk has evolved over time and there are still many variations on how to describe high-risk disease characteristics. Risk stratification is crucial for better understanding of the disease prognosis and rational use of therapies to achieve risk-adapted treatment. Additionally, risk stratification is essential for understanding the risk-based diversity of patients in clinical trials – why do certain patients respond better than others.

Studying the genetics of MM offers more insight into the cancer cells and molecular risk stratification. About 20% of newly diagnosed multiple myeloma (NDMM) patients have molecular abnormalities that account for high risk. Fluorescence *in situ* hybridization (FISH) can detect chromosomal aberrations, such as deletion, translocation and gain. Presence of one these aberrations (*single hit*) have been associated with worse outcomes in MM patients. Furthermore, presence of more (*double or triple hit*) of such aberrations indicate serious genomic instability and a very aggressive disease (6, 7). Gene expression profiling (GEP) has also been introduced as a tool for risk stratification in MM based on the genetic make-up of myeloma cells and offers additional prognostic subgroups (4).

The recent introduction of novel agents with multiple modes of action has primarily benefited patients with standard-risk disease defined by current criteria. Although the treatment outcome of patients with high-risk disease has improved, the unfavorable impact of high-risk FISH abnormalities has not been abrogated. Therefore, this review will focus on the standardized analysis of myeloma cancer cells by GEP and describe how GEP can provide additional insights for optimal risk-adapted treatment of MM patients.

## The Challenge of Defining the High-Risk Patient

One of the controversies that limit risk-adapted treatment in MM is the challenge of defining the high-risk patient. Stratifying patients into different risk groups depends on several aspects. Molecular abnormalities are one way to determine high risk, but the clinical behavior of the patient is another one. In order to identify the group of patients that are not receiving the right treatment, both patient clinical behavior and molecular abnormalities need to be integrated into the definition of high risk.

### Clinical Risk

Patient frailty, renal failure, extramedullary disease, tumor burden, early relapse and minimal residual disease can all predict high-risk disease (8–11). The Durie-Salmon staging system, introduced in 1975, reflects the tumor burden by using immunoglobulin levels, hemoglobin and calcium concentration and the number of bone lesions as the classification criteria (9). Although widely accepted from its time of publication, the Durie-Salmon staging system lacks reproducibility and has problematic performance in patients under treatment (9, 12).

In the following years, the relevance of two important and highly prognostic factors appeared in the clinical field. The first is proliferation rate and disease severity indicated by albumin and its inverse relation with interleukin-6 – a known growth and survival factor of myeloma cells (13, 14). The second is tumor burden and renal function reflected by β-2 microglobulin (15–22). Serum albumin and β-2 microglobulin have shown to be better indicators of prognosis and have outperformed the Durie-Salmon system (23). In 2005, a large international consortium of myeloma key opinion leaders defined a staging system on the basis of 10,750 patients from three continents that was about to be the new standard: the International Staging System (ISS), that based its three-group stratification on a combination of the two most powerful and reproducible markers – albumin and β-2 microglobulin (24). The role of tumor burden is however affected by age. Data from 3894 patients uniformly treated in the Myeloma XI trial shows that ISS plays the major role in older patients when defining the survival risk and is of less importance in younger patients (25).

Early relapse, that is a relapse occurring within 12 months from autologous stem cell transplantation (ASCT), is a marker of high-risk disease. Early relapse is associated with reduced survival even after an intensive first line of treatment (8, 26). The first line of treatment in MM is considered crucial in order to prolong the duration of response and survival (8). In similar fashion, patients who do not achieve long lasting minimal residual disease (MRD) negativity are also considered high-risk (11). MRD negativity has become the new end point in treatment, especially for NDMM. Additionally, highly sensitive MRD monitoring may allow for better prediction of early relapse.

### Molecular Risk

In parallel with defining staging systems on the basis of clinical variables, the role of cytogenetics in multiple myeloma was being investigated. Cytogenetics has shown to play a role in other hematologic malignancies but was difficult to study in MM mainly due to low proliferation of myeloma cells, which hampered karyotyping (27). The emergence of FISH enabled the analysis of genetic aberrations independent from cell cycle phases and thereby allowed research of the prognostic value of single chromosomal abnormalities (28). Primary genetic events involved in MM include immunoglobulin heavy chain gene translocations and hyperdiploidy (29, 30). In general, patients with translocations t(4;14) or t(14;16) are considered high-risk (31–35), whereas patients with t(11;14) are considered standard-risk (31, 32, 36, 37) and have a better prognosis. As MM progresses, secondary genetic aberrations develop including mutations and copy number abnormalities, del(13q) (31, 38–40), del(17p) (31–34), del(1p) and gain of 1q (34, 41–43).

With chromosomal abnormalities obtained through FISH adding information to the prognosis of MM patients, the International Myeloma Working Group proposed the revised ISS (R-ISS) in order to add the presence or absence of cytogenetic markers t(4;14), t(14;16), del(17p) and serum lactate dehydrogenase (LDH) (44). Higher levels of LDH are a proxy for high proliferation rates or the presence of tumor mass leading to extramedullary and extraosseous disease and have shown prognostic value in various treatment settings (45–49).

To further add to the prognostic arsenal of clinical and biological markers, various molecular gene classifiers were developed to stratify patients on the basis of up- and downregulated genes (**Table 1**). In 2007, Shaughnessy et al. reported a 70-gene signature with a 17-gene subset that predicts prognosis and stratifies patients in two risk groups (50). This 70-gene classifier is known as GEP70 or UAMS70 and has the brand name MyPRS. In 2008, Decaux et al. published a 15-gene signature that stratifies patients in low or high-risk group, developed by the Intergroupe Francophone du Myélome and called IFM15 (52). In 2010, Dickens et al. defined a 97-gene signature containing cell death genes and reflecting prognosis. A subset of 6 genes were identified to predict poor prognosis and formed the MRCIX6 gene classifier (53). In 2011, Shaughnessy et al. published GEP80 model, that could identify an additional 9% of fast progressing high-risk patients in the patient group defined low risk by GEP70 (51). Also in 2011, Hose et al. reported a gene expression based proliferation index stratifying patients in a low-, intermediate- and high-risk group for disease progression (54). In 2012, Kuiper et al. defined a 92-gene signature that stratifies patients in a standard and high-risk group (4). This 92-gene classifier, called SKY92, is commercially available under the brand name SKY92 or MMprofiler.

**Table 1:** GEP risk signatures and their characteristics.

Scientific name (Brand name)	Platform	Classification	Utility	Performance*	Availability
UAMS70/GEP70 (50) (MyPRS/MyPRS Plus)	ThermoFisher U133Plus2.0 microarray	Continuous score with a cutoff such that patients are either:	Predicts event-free survival and OS at the moment of diagnosis or at relapse.	High-risk score present in 13% of patients. HR = 5.16 (p<0.001) in training set and HR = 4.75 (p<0.001) in the test cohort.	In research setting only.
		- High risk for disease progression - Low risk for disease progression			
UAMS80/GEP80 (51)	ThermoFisher U133Plus2.0 microarray	Dichotomous score such that patients are either:	Predicts PFS and OS at the moment of diagnosis.	GEP80 identifies 9% of high-risk patients in the GEP70 low-risk group and 41% of low-risk patients in the GEP70 high-risk group.	In research setting only.
		- High risk with significantly inferior PFS and OS - Low risk with significantly better PFS and OS			
IFM15 (52)	Custom designed platform	Dichotomous score such that patients are either:	Predicts OS at the moment of diagnosis.	Survival at 3 years was 90.5% (95% CI, 85.6%-95.3%) for the very-low risk group and 47.4% (95% CI, 33.5%-60.1%) for the high-risk group; as estimates of rates from training, test and external validation cohorts.	In research setting only.
		- High risk with significant shorter OS - Very low risk with significant better OS			
EMC92/SKY92 (4) (MMprofiler)	ThermoFisher U133Plus2.0 microarray	Dichotomous score such that patients are either:	Predicts PFS and OS at the moment of diagnosis or at relapse.	High-risk patients showed reduced OS with HR=3.40 (95% CI, 2.19-5.29) for TT2; 5.23 (95% CI, 2.06-4.39) for TT3; 2.38 (95% CI, 1.65-3.43) for MRC-IX and 3.01 (95% CI, 2.06-4.39) for APEX patient cohort. All with p<0.0001.	Both in research setting and commercially (CLIA validated Laboratory Developed Test in the US and CE-IVD certified in Europe).
		- High risk of early relapse - Standard risk of early relapse			

CI, confidence interval; HR, hazard ratio; OS, overall survival; PFS, progression-free survival.

*Performance as described by the authors in respective discovery papers.

### The Challenge of Defining High Risk in MM

Globally, there is no consensus on the definition of high risk, nevertheless clinical experts agree that it is never a single marker. Furthermore, clinical experts seem to also agree on an escalated treatment paradigm for high-risk patients with prolonged planned maintenance (55–58). The National Comprehensive Cancer Network (NCCN) states “patients with cytogenetically and molecularly defined high-risk disease do not receive the same benefit from certain approaches as the low-risk patients and need alternative therapies”.^2^ The International Myeloma Working Group (IMWG) concludes that “risk stratification in MM is important to predict survival and to define a treatment strategy” (59). In none of the guidelines preferred risk-stratified treatment pathways have been described.

**Figure 1:**
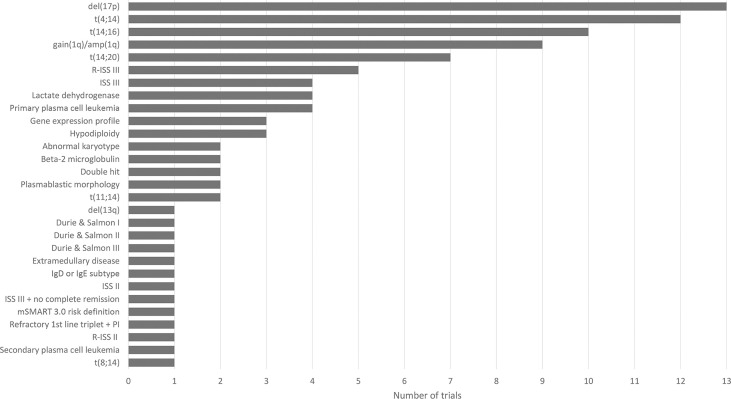
Overview of 29 high-risk markers for multiple myeloma resulting from the analysis of 17 clinical trials (NCT00570180, NCT00691704, NCT00793572, NCT01341262, NCT01668719, NCT02217163, NCT02685826, NCT03004287, NCT03104842 (GMMG-CONCEPT), NCT03188172 (OPTIMUM), NCT03441958, NCT03606577 (IFM 2018-04), NCT03641456, NCT04133636 (CARTITUDE-2), NCT04196491 (KarMMa-4), NCT04579523, NCT04935580). Figure shows the diversity in high-risk marker selection. For each marker, the number of trials selecting the marker to define high-risk disease is shown.

The challenge of defining high risk is also reflected in clinical trials studying the efficacy of treatment (combinations) (**Figure 1**). We have performed a search on August 11, 2021 on ClincialTrials.gov on condition “multiple myeloma” in combination with the terms “newly diagnosed” and “high risk”. The search resulted in 78 studies that were “not yet recruiting”, “recruiting”, “enrolling by invitation”, “active, not recruiting” or “completed”. We further analyzed 17 trials mentioning high-risk as eligibility criteria for enrollment and found 29 different high-risk markers. **Figure 1** lists all 29 markers and shows the diversity in selecting markers to define high risk across clinical trials in MM.

## Gene Expression Profiling Identifies a Unique Group of High-Risk Patients

The developed GEP signatures provide additional insights into risk stratification in a robust manner. The clinical application of the GEP signatures in myeloma has been stagnant because of the lack of standardization and user-friendly platforms (60). Clinical development of SKY92 has overcome these issues, by providing a standardized, analytically validated and user-friendly tool (61). At this moment, the SKY92 signature is the only fully accredited GEP signature and has consistent performance in detecting high risk in MM (61). Therefore, from this section onwards we will focus on SKY92.

### The GEP-Based Marker SKY92

The prognostic GEP-based marker SKY92 was developed as EMC-92 based on the HOVON65/GMMG-HD4 trial using a cohort of 290 NDMM patients. The prognostic power in the combination of 92 genes (including several known cancer genes) was generated by supervised principal component analysis in combination with simulated annealing (4). SKY92 provides a binary outcome and classifies a patient at either high risk with poor survival or at standard risk (61).

SKY92 is clinically validated for accurately predicting the prognosis of NDMM and relapsed/refractory multiple myeloma (RRMM) patients for both overall survival (OS) as well as progression free survival (PFS). After discovery in 2012, SKY92 has been independently validated in 16 patient cohorts totaling 3,339 patient cases (**Table 2**) (73). The validation cohorts cover a wide variety of geographies and treatments, clinical trial and non-trial real world settings, and both transplant eligible as well as non-transplant eligible patients. The proportion of high-risk patients identified by SKY92 remains stable around 20% in the NDMM validation sets and is slightly higher for the poorer performing RRMM patients.

**Table 2:** SKY92 clinical validation studies.

Cohort	MM type*	N	SKY92 high risk (%)	Hazard ratio OS (p-value)	Hazard ratio PFS (p-value)
**HOVON-65/GMMG-HD4 (** 4 **)**	ND	329	–		
**TT2 (** 4 **)**	ND	351	68 (19%)	3.4 (<0.0001)	
**APEX (** 4 **)**	RR	264	43 (16%)	3.0 (<0.0001)	1.7 (0.0058)
**TT3 (** 62 **)**	ND	254	47 (19%)	4.5 (<0.0001)	
**MMGI (** 63 **)**	ND	91	19 (21%)	8.2 (<0.0001)	
**GIMEMA-MMY-3006 VTD (** 64 **)**	ND	114	23 (20%)	4.0 (0.0037)	
**CoMMpass (** 65 **)**	ND	632	116 (18%)	3.1 (<0.0001)	
**HOVON-87/NMSG-18 (** 66 **)**	ND	190	26 (14%)	2.6 (<0.0001)	2.4 (<0.0001)
**KRd trial (** 67 **)**	ND	16	5 (31%)		8.2 (0.017)
**CarThaDex trial (** 68 **)**	ND	20	5 (25%)		2.8 (0.12)
**EMN-02/HOVON-95 (** 69 **)**	ND	179	36 (20%)		
**E-MTAB-1038 (** 70 **)**	ND/RR	66	13 (20%)	2.6 (0.044)	
**TT6 (** 70 **)**	RR	55	11 (20)	10.3 (0.00015)	
**MMpredict non-trial set (** 71 **)**	ND/RR	155	34 (22%)	4.5 (<0.0001)	2.7 (<0.0001)
**MUKseven trial (** 72 **)**	RR	48	9 (25%)		2.9 (0.037)
**MRC-IX (** 34 **)**	ND	246	51 (21%)	2.2 (<0.0001)	
**MRC-XI (** 34 **)**	ND	329	81 (25%)	3.9 (<0.0001)	2.6 (<0.0001)
**Total**		3,339	587		

*ND, newly diagnosed; RR, relapsed/refractory.

This table has been published by Biran et al. (73) and can be reproduced under the terms of Creative Commons Attribution 4.0 license, https://creativecommons.org/licenses/by/4.0/.

Longitudinal analysis of two publicly available MM patient data sets aimed to investigate the evolution of SKY92 risk classification with disease progression (74). SKY92 risk classification was compared between same-patient samples at diagnosis and relapse in the Multiple Myeloma Research Foundation (MMRF) CoMMpass data set and the University of Arkansas for Medical Sciences (UAMS) Total Therapy cohort (TTx). In the analysis of the CoMMpass data set, 31% of patients were classified high-risk at diagnosis. The number of high-risk patients increased to 46% at the second timepoint and 58% for patients with the latest timepoint more than 12 months after baseline. Furthermore, almost all patients in the CoMMpass data set died within 12 months after being classified as high-risk. In the TTx cohort the percentage of high-risk patients increased significantly from 12% at diagnosis to 28% at relapse. Analysis of these data sets show that repeated testing of risk signature provides additional prognostic information for standard-risk patients.

The PROMMIS trial (NCT02911571) investigated the impact of SKY92 on risk stratification and treatment plan (73). This prospective observational study showed that SKY92 added more information on risk stratification, compared to currently used standards. In the PROMMIS trial, physicians first classified the MM patients according to their own local standard and determined the patient’s treatment path accordingly. Then, after unblinding SKY92 results, physicians had the opportunity to reclassify the patients and adapt treatment. Overall, Unblinding SKY92 results led to a change of risk status for 42% of patients (62/147). More specifically, 16 patients received a SKY92 high-risk result while previously being assigned standard risk – all of these patients (100%) were reclassified high risk after unblinding SKY92; 46 patients received a SKY92 standard-risk result while previously being assigned high risk – 30 out of 46 (65%) were reclassified standard risk after unblinding SKY92. Treatment plans were changed for 37% of patients (54/147). After knowing SKY92 results, physicians were more confident in their final treatment plan, even when SKY92 confirmed their initial risk classification. For 89% of patients (131/147), the final risk classification assigned by the physician matched the SKY92 result, showing the added value of SKY92 in clinical decision making.

### Combining GEP-Based Biomarkers With (R-)ISS

Besides the use of univariate prognostic markers such as (R-)ISS, single cytogenetic abnormalities or single gene expression classifiers, the combination of prognostic markers is being increasingly investigated (34, 75). This allows for more specific risk classifications, shifting from the ability to determine if a patient is high risk (and otherwise standard risk) towards a multi-categorical classification.

In a multivariate analysis, 20 clinical and biological risk markers were used independently to find the strongest predictor for prognosis as either a stand-alone marker or in combination (62). A total number of 4,750 patients were included from the APEX, HOVON65/GMMG-HD4, IFM-G, MRC-IX, TT2 and TT3 cohorts. The research showed that ISS is a valuable partner to both GEP classifiers and FISH markers. Ranking all existing and combined risk markers showed that GEP+ISS is the strongest predictor for OS, resulting in a 4-group risk classification. In this setting GEP, by means of SKY92, stratified patients into the high-risk and the standard-risk group. ISS sub-stratified the standard-risk patients into two intermediate-risk groups (ISS II + ISS III) and a low-risk group (ISS I group). The median survival was 24 months for the high-risk group, 47 and 61 months for the intermediate-risk groups, and the median survival was not reached after 96 months for the low-risk group.

The GEP classifier SKY92 has also been combined with R-ISS in the HOVON-87/NMSG-18 trial in an analysis of 168 older myeloma patients (76). Combining the R-ISS with SKY92 resulted in 3 risk groups: SKY-RISS I (SKY92 standard risk + R-ISS I, 15%), SKY-RISS III (SKY92 high risk + R-ISS II/III, 11%) and SKY-RISS II (all other patients, 74%). The 3-year OS rates for SKY-RISS I to III were 88%, 66% and 26% (p=6x10^-7^) and were validated in the elderly patient subset from the CoMMpass dataset. Combining SKY92 with R-ISS resulted in a superior prognostic marker compared to either marker separately.

### Unique Group of High-Risk Patients Is Identified With GEP

In order to make sure that a high-risk patient is correctly identified, newer, more robust, standardized and reliable technologies should be incorporated for risk stratification. There is substantial evidence that GEP is indispensable for correct assessment of high-risk MM patient population after which the well-established clinical and cytogenetic markers can further distinguish the low-risk from the intermediate-risk patients.

In 2019, Kuiper et al. analyzed PFS and OS related to high-risk outcomes based on ISS and SKY92 in non-transplant eligible patients from the HOVON87/NMSG18 study (66). In this cohort, 26% of patients were classified as high risk by ISS (ISS III). SKY92 classified 14% of patients as high-risk, with an overlap between the two groups of 5%. Thus, 9% of high-risk patients are misclassified as lower risk (ISS I or ISS II). As the R-ISS is becoming the preferred staging system, in 2020 Kuiper et al. compared prognostication between R-ISS and SKY92 in older myeloma patients (76). In the HOVON87/NMSG18 cohort, R-ISS III identified 7% of patients as high risk, where SKY92 identified 13%. Furthermore, in the CoMMpass cohort these percentages were 13% and 26%, respectively.

Several multivariate analyses have established that SKY92 is an independent prognostic marker and that combining SKY92 with ISS or R-ISS results in a marker with improved performance compared to either marker separately (34, 66, 76). But how does the prognostic power of SKY92 relate to several high-risk cytogenetic markers such as del(17p) and t(4;14)? Combining six datasets, for a total of 805 patients, SKY92 combined with ISS identified three risk groups: low, intermediate and high (77). The OS of the high-risk group was significantly shorter than the low-risk group (hazard ratio 6.0, p<0.001). For all three risk groups, the comparison of FISH-positive and FISH-negative patients resulted in a non-significant OS difference. In the high-risk group (n=169), 53% of patients (90/169) were FISH-negative. The high-risk status of these 90 patients was overlooked by using only FISH for risk stratification.

In 2020, Shah et al. examined the combined predictive value of high-risk chromosomal abnormalities, including t(4;14), t(14;16), t(14;20), gain(1q) and del(17p), and SKY92 in 329 NDMM patients from the NCRI Myeloma XI trial who received intensive therapy and validated the findings in Medical Research Council (MRC) Myeloma IX trial (34). SKY92 identified 24.6% high-risk patients (81/329) with a significantly shorter PFS (median 16 versus 33.8 months; hazard ratio 2.6, CI 95% 2.0-3.5; p=4.1x10^-11^) and OS (median 36.7 months versus not reached; hazard ratio 3.9, 95% CI: 2.7-5.7; p=2.5x10^-13^), regardless of induction regimen and posttransplant randomization. There was a partial overlap between patients with SKY92 and chromosomal high-risk markers, with 6.1% (20/329) of patients identified as SKY92 high risk in the absence of chromosomal high-risk markers. Furthermore, 161 patients carried no chromosomal high-risk marker, of which 12% (20/161) were SKY92 high risk. These 20 patients had significantly shorter PFS (median 15.8 versus 41.7 months; hazard ratio 3.18, 95% CI: 1.86-5.46; p=2.6x10^-5^) and OS (estimated 4-year OS 55% versus 86%; hazard ratio 2.42, 95% CI: 1.04-5.67; p=0.04). The study demonstrated the prospective prognostic validity of SKY92 in the wider context as a means of identifying patients at diagnosis who have high-risk MM. Furthermore, the study highlighted that SKY92 combined with chromosomal profiling at diagnosis can predict clinical outcome with significant precision. The authors of the study also acknowledge that the identification of high-risk patients opens up the possibility of risk-adapted treatment.

UK OPTIMUM trial (MUKnine, NCT03188172) is a prospective study from 39 UK hospitals designed to identify ultra high-risk patients and provide risk-adapted treatment. Patients were centrally profiled for GEP high-risk signature and/or double hit disease. These patients were considered ultra high-risk and were treated with daratumumab, cyclophosphamide, bortezomib, lenalidomide, dexamethasone (Dara-CVRd) induction, augmented high-dose melphalan and ASCT (78). MRD status was 64% MRD-negative, 14% MRD-positive and 22% not evaluable at day 100 post ASCT (assessed by flow cytometry, sensitivity 10^-5^). Despite overall high MRD-negativity, some early progressions indicate a group of patients with unmet clinical need. Furthermore, OPTIMUM trial is a digital comparator arm trial. PFS at 18 months was compared between patients in the OPTIMUM trial and matched ultra high-risk myeloma patients from the Myeloma XI trial treated with carfilzomib, lenalidomide, cyclophosphamide and dexamethasone, ASCT and lenalidomide maintenance or observation. Patients from the OPTIMUM trial, treated with the five-drug combination, were found to have significantly improved PFS; with an 18 months estimate of 81.7% (95% CI: 74.2-89.1) and 65.9% (95% CI: 57.3-74.4) for the OPTIMUM and Myeloma XI trial, respectively (79). The analyses of the OPTIMUM trial have shown that identifying the ultra high-risk patients and adapting the treatment can lead to high MRD negativity rates and improved survival in comparison to the standard of care. Lastly, OPTIMUM trial demonstrated the feasibility of incorporating GEP into clinical patient pathway across institutions and showed the value of combining cytogenetic information with GEP.

## Discussion

Several new drugs have been added to the therapeutic landscape of MM in recent years, which have contributed to increased PFS and OS. However, while the use of therapeutics in the clinical scenario has evolved, there is still a group of high-risk patients who do not benefit from current treatment strategies. The international guidelines on MM all recognize the relevance of having detailed information on the patient’s disease and associated risk for progression in order to better evaluate the individual clinical care pathway (59). It has become clear that there is not a single marker capable of independently and accurately defining the high-risk MM patient. For this reason, multiple combinations are postulated by different study groups, but no real consensus has been formulated and used in clinical trials.

In this review, we aimed to describe the impact of the genetic make-up of cancer cells as a molecular risk factor in MM. In this section, we will discuss the integration of both clinical variables and molecular markers into the definition of high risk and the outlook of risk-adapted treatment in MM.

GEP-based marker SKY92 is a standardized tool for risk stratification that provides additional information to the anamneses of patients with MM. SKY92 allows for risk stratification in relatively homogenous subgroups of patients and provides added value in combination with clinical markers and FISH, which by itself does not capture the genomic complexity of MM (27, 34). SKY92 stratifies MM patients into high-risk or standard-risk group irrespective of treatment regime and relapse setting. Risk stratification using SKY92 is important at diagnosis in order to choose the optimal first line of treatment for maximum effect and prevention of relapse.

Longitudinal monitoring of risk assessment using SKY92 allows for dynamic risk stratification (74). GEP in combination with FISH is a great way to track changes in molecular risk over time and should be performed at diagnosis and every relapse to correctly identify high-risk patients and guide treatment (80, 81).

Risk can also change during the course of disease depending on response to treatment. Early relapse has strong association with reduced survival in both high-risk and standard-risk cytogenetic groups (8). MRD also plays a big role in patient prognosis. MRD-negative patients with high-risk cytogenetics have similar outcome as standard-risk patients (82). However, further research is needed to investigate the proper actions that should be taken regarding de-escalation of therapy of patients with sustained MRD-negativity.

There are still some limitations of using GEP in practice. Bone marrow sample availability is one of the challenges, as well as the lack of guidelines for optimal risk classification and corresponding treatment strategy. Additionally, SKY92 was developed and validated for active and symptomatic MM, therefore the prognostic value of SKY92 has yet to be assessed in (asymptomatic) smoldering myeloma. Lastly, a cost-benefit analysis of SKY92 still needs to be performed because current evidence is lacking. Such analysis should be done to investigate the cost impact of SKY92 on the use of high-cost anti-myeloma drugs.

Despite the current limitations, there is substantial evidence that GEP is an important tool in providing the most accurate risk assessment identifying the true high-risk population and can be used in combination with the well-established clinical and cytogenetic markers. After GEP risk assessment, ISS and FISH can further distinguish the intermediate-risk from the low-risk patients. Without GEP, many patients are misclassified using existing tools. Therefore, a new era beckons in which patients are routinely and accurately assigned risk and its relevance, considering the available treatment opportunities. In this new era:

- GEP is used to identify high-risk MM patients;
- GEP is part of routine diagnostic work-up to allow for risk-adapted strategies;
- Risk-adapted treatment is investigated in both clinical trials and the real-world setting.

## Concluding Remarks and Future Perspectives

With the availability of new techniques and increasing knowledge of MM biology, the definition of high risk is evolving and should include personalized assessment of both clinical and molecular markers. Treatment should focus on biology of high risk in younger patients and on clinical behavior in older patients. Treatment intensification in molecular high-risk patients and dose reduction and deliverability in clinical high-risk and frail patients need to be researched.

Treatment combining several modes of action and incorporation of novel immunotherapies, for example CAR T-cell therapy or bispecific antibodies (83), could be the next area to explore for the high-risk patients that represent the group with unmet clinical need. Risk-adapted therapy is crucial to achieve deep and sustained response in high-risk MM patients. In order to develop therapeutic strategies for specific risk groups, it is of utmost importance to use GEP as an eligibility marker. In many trials the risk groups are stratified along different randomization arms or inclusion criteria. For both misclassified high-risk patients in a low-risk trial and low-risk patients in high-risk trials, the final conclusions on the effectiveness of the investigated therapeutic regimen will be influenced. Clinical trials focusing on high-risk MM patients are crucial for identification of optimal therapy for improved survival.

## Author Contributions

CC wrote the manuscript. AKS, LR, and PS reviewed and edited the manuscript. SU, MKa, MKo, MVM, AS, and KA reviewed the manuscript. All authors contributed to manuscript revision, read, and approved the submitted version.

## Conflict of Interest

AKS has received consulting fee from SkylineDx. SU and PS have received research funding and consulting fees from SkylineDx.

The remaining authors declare that the research was conducted in the absence of any commercial or financial relationships that could be construed as a potential conflict of interest.

## Publisher’s Note

All claims expressed in this article are solely those of the authors and do not necessarily represent those of their affiliated organizations, or those of the publisher, the editors and the reviewers. Any product that may be evaluated in this article, or claim that may be made by its manufacturer, is not guaranteed or endorsed by the publisher.

## Footnotes

1 GLOBOCAN database from the International Agency for Research on Cancer (IARC). Available at: https://gco.iarc.fr. Accessed July 30, 2020.

2 Kumar SK. et al. NCCN Clinical Practice Guidelines in Oncology (NCCN Guidelines) Multiple Myeloma Version 4.2020 – May 8, 2020. Available at: https://nccn.org. Accessed August 3, 2020.
